# Cannabis dispensary staff approaches to counseling on potential contraindications to cannabis use: insights from a national self-report survey

**DOI:** 10.1186/s12875-023-02095-5

**Published:** 2023-07-14

**Authors:** Deepika E. Slawek, Andrew D. Althouse, Robert Feldman, Julia H. Arnsten, Hailey W. Bulls, Jane M. Liebschutz, Shannon M. Nugent, Steven R. Orris, Rebecca Rohac, Joanna L. Starrels, Benjamin J. Morasco, Devan Kansagara, Jessica S. Merlin

**Affiliations:** 1 Division of General Internal Medicine, Albert Einstein College of Medicine, Bronx, NY USA; 2 Montefiore Medical Center/Albert Einstein College of Medicine, 111 E 210th St, Bronx, NY 10467 USA; 3 Division of General Internal Medicine, Center for Research on Health Care, University of Pittsburgh, Pittsburgh, PA USA; 4 CHAllenges in Managing and Preventing Pain (CHAMPP) Clinical Research Center, University of Pittsburgh, Pittsburgh, PA USA; 5 Department of Psychiatry, Oregon Health and Science University, Portland, OR USA; 6 Center to Improve Veteran Involvement in Care, VA Portland Health Care System, Portland, OR USA; 7 Department of Medicine, Oregon Health and Science University, Portland, OR USA

**Keywords:** Cannabis, Medication safety, Medical cannabis, Health policy

## Abstract

**Background:**

Legal cannabis is available in more than half of the United States. Health care professionals (HCPs) rarely give recommendations on dosing or safety of cannabis due to limits imposed by policy and lack of knowledge. Customer-facing cannabis dispensary staff, including clinicians (pharmacists, nurses, physician’s assistants), communicate these recommendations in the absence of HCP recommendations. Little is known about how dispensary staff approach individuals with complex medical and psychiatric comorbidities. Using responses from a national survey, we describe how cannabis dispensary staff counsel customers with medical and psychiatric comorbidities on cannabis use and examine whether state-specific cannabis policy is associated with advice given to customers.

**Methods:**

National, cross-sectional online survey study from February 13, 2020 to October 2, 2020 of dispensary staff at dispensaries that sell delta-9-tetrahydrocannabinol containing products. Measures include responses to survey questions about how they approach customers with medical and psychiatric comorbidities; state medicalization score (scale 0-100; higher score indicates more similarity to regulation of traditional pharmacies); legalized adult-use cannabis (yes/no). We conducted multiple mixed effects multivariable logistic regression analyses to understand relationships between state medicalization and dispensary employees’ perspectives.

**Results:**

Of 434 eligible respondents, most were budtenders (40%) or managers (32%), and a minority were clinicians (18%). State medicalization score was not associated with responses to most survey questions. It was associated with increased odds of encouraging customers with medical comorbidities to inform their traditional HCP of cannabis use (Odds ratio [OR]=1.2, 95% confidence interval [CI] 1.0-1.4, *p*=0.03) and reduced odds of recommending cannabis for individuals with cannabis use disorder (CUD) (OR=0.8, 95% CI 0.7-1.0, *p*=0.04). Working in a state with legalized adult-use cannabis was associated with recommending traditional health care instead of cannabis in those with serious mental illness (OR 2.2, 95% CI 1.1-4.7, *p*=0.04). Less than half of respondents believed they had encountered CUD (49%), and over a quarter did not believe cannabis is addictive (26%).

**Conclusions:**

When managing cannabis dosing and safety in customers with medical and psychiatric comorbidity, dispensary staff preferred involving individuals’ traditional HCPs. Dispensary staff were skeptical of cannabis being addictive. While state regulations of dispensaries may impact the products individuals have access to, they were not associated with recommendations that dispensary staff gave to customers. Alternative explanations for dispensary recommendations may include regional or store-level variation not captured in this analysis.

**Supplementary Information:**

The online version contains supplementary material available at 10.1186/s12875-023-02095-5.

## Background

The United States is experiencing a rapid increase in cannabis legalization. As of February 2023, 37 states including the District of Columbia have legalized medical cannabis use, 21 of which also legalized adult-use (recreational) cannabis [1–3]. Most individuals who purchase medical or adult-use cannabis at dispensaries intend to use it for therapeutic purposes [4–6]. Cannabis is used for medical and psychiatric conditions to manage symptoms such as pain, nausea, anxiety, insomnia, or cachexia [4, 7, 8].

Evidence suggests that individuals primarily receive guidance on cannabis use from customer-facing dispensary staff (people who sell cannabis at dispensaries [ sometimes referred to as “budtenders”]; managers; or dispensary clinicians such as pharmacists, physicians, nurse practitioners, or physician assistants), rather than from traditional healthcare providers [9]. In most US states with legalized medical cannabis, health care professionals (physicians and advanced practice providers such as nurse practitioners and physicians’ assistants) “certify” that an individual meets state criteria for medical cannabis, but they are typically not required to and often do not identify a specific product or dosing recommendation that should be purchased at the dispensary or specify dosing recommendations in the same way that other prescribed medications are. Therefore, dispensary staff guide individuals on cannabinoid dose, ratio of delta-9-tetrahydrocannabinol (THC) to cannabidiol (CBD) and administration. Few traditional healthcare professionals receive training in cannabis or the endocannabinoid system, and many feel ill-equipped to counsel individuals on cannabis use, safety, adverse effects, and drug interactions [10–14]. This is likely due to a paucity of research on therapeutic cannabis use. Systemic barriers, such as its status as a Schedule I substance by the U.S. Controlled Substance Act [15], have resulted in limited empirical literature about its use.

Cannabis safety and appropriateness are impacted by medical comorbidities and other factors. For example, cannabis that has high doses of THC can trigger or exacerbate psychosis in individuals with predisposition for psychotic spectrum disorders [16]. Cannabinoids, metabolized through the cytochrome P450 system, impact the metabolism of medications that use the same system, such as statins and warfarin. Cannabinoids can also have an additive effect or adverse interactions with medications that are sedating [17]. Moreover, 8-12% of those using cannabis will develop cannabis use disorder (CUD) over time [18, 19].

Each state approaches medical cannabis legalization with different degrees of regulation, referred to as ‘medicalization’ [20]. Medical cannabis programs vary by state and are considered more ‘medicalized’ if they have more stringent rules such as: mandatory cannabinoid content and contaminant testing, mandatory warning labels about potential hazards or health effects, standardized training of dispensary staff on cannabis, or mandatory protocols for handling individuals with contraindications to cannabis use at dispensaries. Thus, medicalization may be associated with dispensary practices, especially when interacting with individuals with medical and psychiatric comorbidities. However, the extent of these disparities is unknown.

Given the role customer-facing dispensary staff play in the recommendations on how to use cannabis, this study investigates how they counsel individuals with medical and psychiatric comorbidities (including CUD) about cannabis use. We hypothesized that dispensary staff working in states with more medicalized cannabis laws, and those with legalized adult-use, would recommend involvement of traditional healthcare professionals and recommend against cannabis use for individuals with comorbidities and CUD more often than dispensary staff working in states with less stringent cannabis laws.

## Methods

### Overview

We present data from a one-time, self-report survey disseminated to cannabis dispensary staff across 34 states from February 13, 2020 to October 2, 2020. The survey development, sampling strategy and recruitment were described in detail elsewhere, and other results have been published separately [21]. Briefly, a group of seven experts in the fields of primary care, addiction medicine, medical cannabis, and behavioral science developed survey questions based on clinical experience and the current literature. Once all members of the group agreed upon the content and phrasing of the questions, dispensary industry contacts piloted the survey, and it was revised iteratively. The final survey asked questions regarding how cannabis dispensary staff counsel customers who have medical and psychiatric comorbidities and reasons for which they have advised against cannabis. We grouped response options into categories e.g., attitudes about cannabis benefits/risks, observations about customers’ cannabis use and attitudes, basis for customer advice. See Supplementary Table 1a for survey questions. This study was considered exempt by the University of Pittsburgh Institutional Review Board and study procedures were performed in accordance with the Declaration of Helsinki.

### Sampling strategy and recruitment

We identified a list of cannabis dispensaries through internet searches of state databases, relevant websites (Leafly.com, weedmaps.com), and a list of 4,715 dispensaries purchased from a marketing company [21]. We recruited respondents by 1) mailing copies of the survey which included instructions for online completion, 2) approaching two national dispensary chains and a cannabis retailer’s association, 3) calling dispensaries and requesting that managers distribute the survey to staff.

### Eligibility criteria

We defined cannabis dispensaries as stores that sold THC-containing products on site. Eligibility for completing the survey included: 1) working at a cannabis dispensary, and 2) interaction with cannabis dispensary customers in the role of providing advice about cannabis purchases.

A response was excluded if: 1) it originated from a state where THC-containing products were illegal at the time of the survey, 2) the respondent worked at a pharmacy or store where THC-containing products were not sold, or 3) the survey was <95% complete. Multiple individuals from the same dispensary were eligible to complete the survey.

### Survey administration

The survey was conducted online via Qualtrics. Respondents received their choice of a one-time $10 payment card immediately after completing the survey or entry into a lottery to receive a $250 payment card at the end of the study as compensation for completing the survey.

### Key variables

#### Dependent variables

This analysis focused on responses to five survey questions, each of which was treated as a dependent variable:

1. *What do you do when you encounter a customer who is using cannabis to treat a medical condition (such as cancer, HIV/AIDS, or multiple sclerosis)?*
2. *What do you do when you encounter a customer who is using cannabis to treat depression, anxiety, or post-traumatic stress disorder?*
3. *What do you do when you encounter a customer who is using cannabis to treat a serious mental illness (such as schizophrenia, bipolar disorder, or psychosis)?*
  For questions #1-3, respondents could answer ‘yes’ or ‘no’ to any of the eight potential responses: (a) encourage customer to inform physician or other healthcare professional about cannabis use; (b) encourage customer to seek traditional medical care in addition to cannabis; (c) encourage customer to do additional research online; (d) encourage customer to seek traditional medical care instead of cannabis; (e) I don’t do anything differently; (f) encourage customer to continue only cannabis; (g) I have never encountered a customer who is using cannabis to treat this (medical, mental health) condition; (h) Other, please describe. For analytic purposes, we also present a composite response of “Encourage customer to seek traditional medical care in addition to OR instead of cannabis” that is considered a “positive” if the respondent answered affirmatively to either (b) or (d) from this list.
4. *What do you do when you encounter a customer who you suspect has a cannabis use disorder?*
  For question #4, respondents could answer ‘yes’ or ‘no’ to any of the six potential responses: (a) I have never encountered a customer who I suspected had CUD; (b) discuss purchasing cannabis products that may help with the CUD; (c) I do not believe cannabis is addictive; (d) refer to a physician or other healthcare professional; (e) I do not do anything differently for such customers; (f) I have never heard of CUD.
5. *Select all reasons why you have advised against cannabis purchase*.
  For question #5, respondents could select ‘yes’ or ‘no’ to a list of 14 responses: (a) pregnancy or nursing; (b) serious mental illness (schizophrenia/bipolar disorder/psychosis); (c) customer appeared intoxicated; (d) customer having legal problems related to cannabis; (e) customer having difficulty affording cannabis; (f) anxiety; (g) cognitive impairment (e.g. dementia); (h) customer needs more cannabis for the same effect; (i) customer was an older adult (age>65 years); (j) customer having difficulty keeping a job; (k) customer has withdrawal symptoms; (l) depression; (m) post-traumatic stress disorder; (n) customer having relationship problem (e.g. with partner or other close family or friends); (o) Other, please describe. Response options (d), (h), (j),(k) and (n) reflect CUD criteria [22].

#### Independent variables

**Fig. 1:**
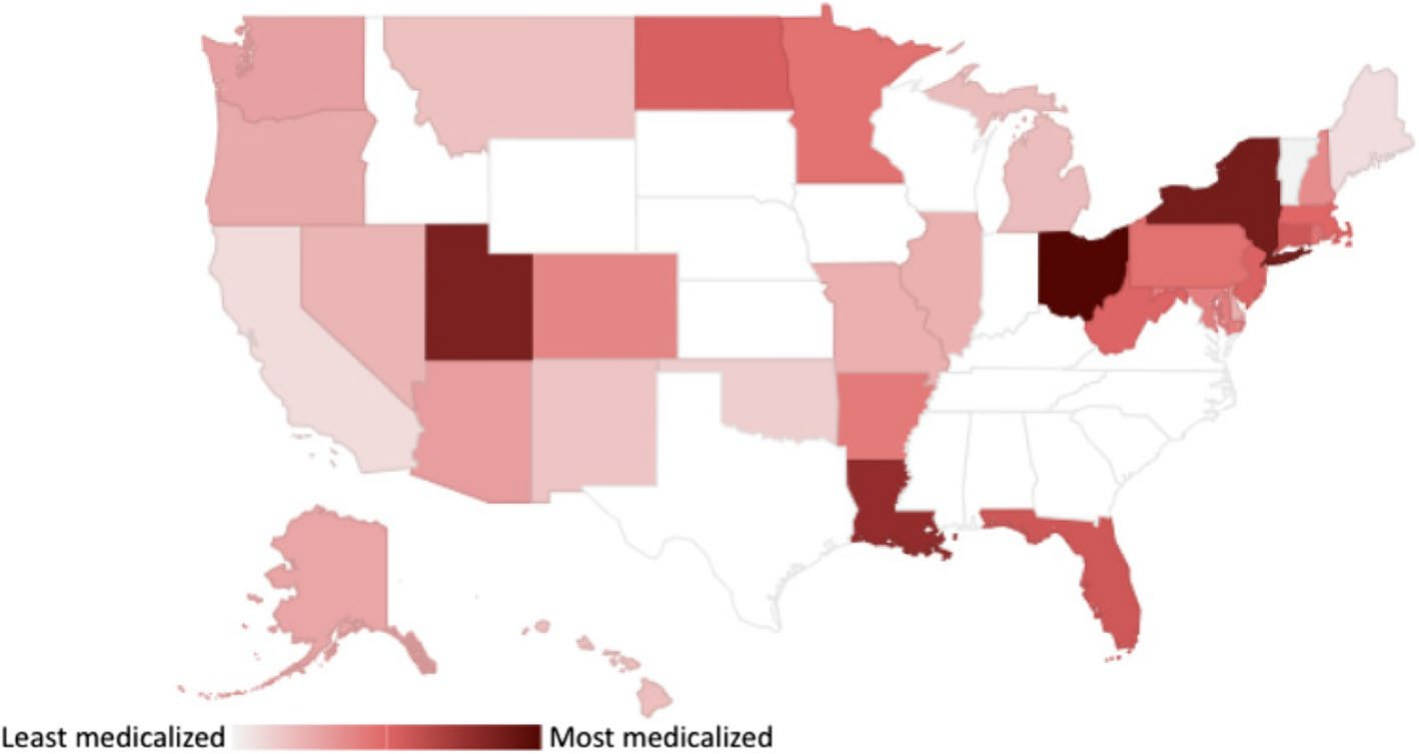
MCLaSS summary score by state. Printed with permission by Elsevier. Originally printed in Richard EL, Althouse AD, Arnsten JH, et al. How medical are states' medical cannabis policies?: Proposing a standardized scale. *Int J Drug Policy.* 2021;94:103202

We considered two independent variables: state medicalization score and legalized adult-use cannabis. State medicalization score is a measure of the extent to which state laws govern the use of medical cannabis similarly to how pharmaceutical medications are governed. This was assessed with the Medicalization of Cannabis Laws Standardized Scale (MCLaSS) [20]. The MCLaSS is a weighted average of scores in seven domains: patient-clinician relationship, manufacturing and testing, product labeling, types of products, supply and dose limit, prescription drug monitoring program, and dispensing practices. We calculated scores for each respondent’s state (range 23 [least medicalized]-86 [most medicalized]) based on cannabis laws in place in July 2019. This timeframe reflected the time needed for laws to impact behavior, especially in the context of the COVID-19 pandemic. A map depicting MCLaSS scores across the entire US is presented in Fig. 1.

Legalized adult-use cannabis was a dichotomous variable indicating whether a state had legalized recreational cannabis as of July 2019.

#### Covariates

Covariates were: age of respondent (years) in 10-point increments, role of respondent (categorical; budtender, manager, other), years working in the cannabis industry (categorical; <6 months, 6 months-1 year, 1-2 years, >2 years, no response), works on sales commission (categorical; yes, no, no response), and education (categorical; completed high school/general educational development test or less, some college or Associate’s Degree, completed 4-year college degree, some graduate school, completed graduate school, prefer not to answer). Other measures were gender (categorical; male, female, other/no response) and length of time in current position (categorical; <6 months, 6 months-1 year, 1-2 years, >2 years, no response).

### Statistical analysis

We present descriptive characteristics as mean (SD) for continuous variables and frequency (percentage) for categorical variables. Multivariable logistic regression models assessed the relationships between the predictor variables of interest (state medicalization score and legalized adult-use cannabis) and the responses to the five survey questions listed above. We conducted separate multivariable analyses for each possible response to each of the five survey questions. Mixed effects models were used, including a random effect for state, to account for clustering since all responses from the same state have the same value for medicalization score / adult use. As affirmatively choosing one response did not preclude choosing the other responses for that question, and we were most interested in understanding the relationship between the independent variables and each response option, we considered each response option independently and did not look for clustering of response options. We did not adjust for multiple comparisons because we determined this to be impractical. Further adjusting for multiple comparisons risked causing a type II error and over-simplifying the relationship between state medical cannabis regulations and practices in cannabis dispensaries [23]. Each full model included age (per 10-point increment), role of respondent, years working in the cannabis industry, collecting sales commission and education as well as state medicalization score and legalized adult-use cannabis.

Due to concerns that clinicians and non-clinicians may have different patterns of responses, we conducted sensitivity analyses limited to non-clinicians only. All statistical analyses were performed using R version 3.6.0.

## Results

**Fig. 2:**
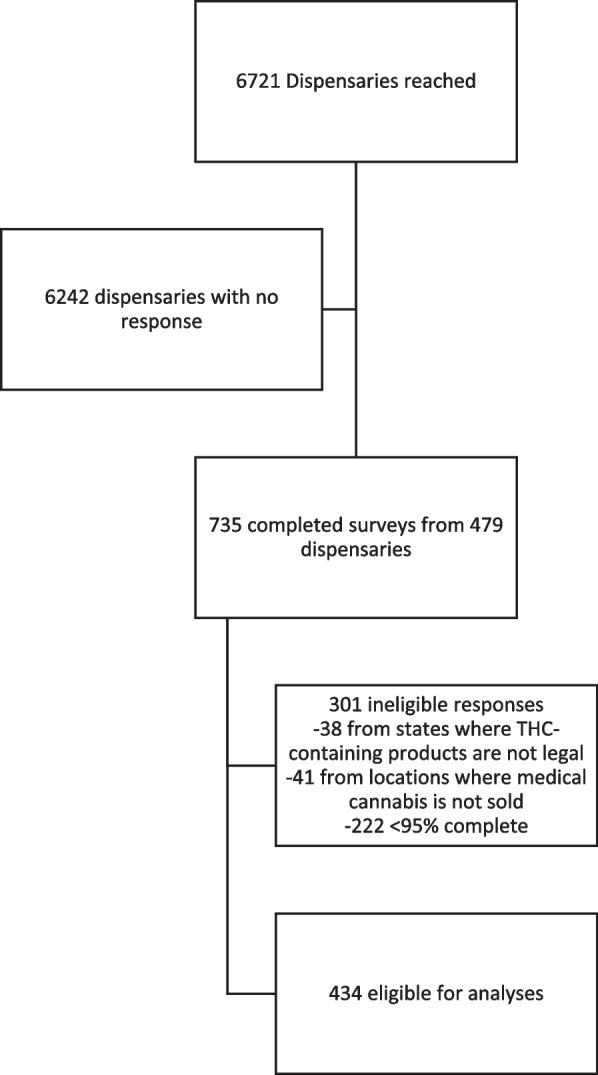
Flowchart of survey recruitment, enrollment, and analysis

We attempted outreach to 6721 dispensaries. Four-hundred seventy-nine (479; 7.1%) returned at least one completed survey. We received 735 responses; 434 were eligible for analysis and 301 were ineligible (222 not complete, 38 from states where THC-products were not legal, 41 from locations where medical cannabis is not sold) (Fig. 2). Respondents were a mean age of 33.4 (standard deviation [SD]= 9.8) and about half were female (51.8%). A majority identified as “budtenders” (39.9%) or managers (32.3%). A minority identified as clinicians, including pharmacists (13.1%) and physicians, nurse practitioners, or physician assistants (5.1%). Half had worked in the cannabis industry for at least 2 years (50.5%) and 39.9% had been in their current position for more than two years. Mean state medicalization score of respondents was 47.6 (SD=15.7). Number of responses from each state ranged from 1-48. States with the least number of responses were Hawaii, Maine, New Hampshire, and Utah. States with the most responses were New York, Oklahoma, and Oregon. Over one-third of respondents worked in a state in which adult-use cannabis was legalized (37.6%) (Table 1).

**Table 1:** Demographics of respondents

	All n (%) *n*=434	Clinicians n (%) *n*=79 (18%)	Non-clinicians n (%) *n*=355 (82%)
Age, years, mean (SD)	33.4 (9.8)	35.1 (9.9)	33.0 (9.8)
**Gender**			
Female	225 (51.8%)	43 (54.4%)	182 (51.3%)
Male	196 (45.2%)	34 (43%)	162 (45.6%)
Other/No Response	13 (3%)	2 (2.5%)	11 (3.1%)
**Role**			
Budtender	173 (39.9%)	--	173 (48.7%)
Manager	140 (32.3%)	--	140 (39.4%)
Physician/NP/PA	22 (5.1%)	22 (27.8%)	--
Pharmacist	57 (13.1%)	57 (72.2%)	--
Other	41 (9.4%)	--	41 (11.5%)
No response	1 (0.2%)	--	1 (0.3%)
Years working in cannabis industry			
<6 months	25 (5.8%)	3 (3.8%)	22 (6.2%)
6 mo to 1 yr	68 (15.7%)	6 (7.6%)	62 (17.5%)
1-2 yrs	116 (26.7%)	28 (35.4%)	88 (24.8%)
>2 yrs	219 (50.5%)	42 (53.2%)	177 (49.9%)
No response	6 (1.4%)	0 (0%)	6 (1.7%)
Length of time in current position			
<6 months	26 (6%)	3 (3.8%)	23 (6.5%)
6 mo to 1 yr	117 (27%)	11 (13.9%)	106 (29.9%)
1-2 yrs	117 (27%)	29 (36.7%)	88 (24.8%)
>2 yrs	173 (39.9%)	36 (45.6%)	137 (38.6%)
No response	1 (0.2%)	0 (0%)	1 (0.3%)
State medicalization score, mean (SD)	47.6 (15.7)	53.2 (19.3)	46.3 (14.5)
Legalized statewide adult use	163 (37.6%)	26 (32.9%)	137 (38.6%)

*Abbreviations*: *SD* standard deviation, *NP* nurse practitioner, *PA* physician’s assistant

Table 2 summarizes participants’ self-reported approach to customers using cannabis to treat conditions such as cancer, HIV/AIDS, or multiple sclerosis. The most frequently selected responses were ‘encourage customer to inform physician or other healthcare professional about cannabis use’ (67.5%), ‘encourage customer to seek traditional medical care in addition to cannabis’ (49.8%), and ‘encourage customer to do research online’ (48.8%). More than half (56.9%) of respondents checked at least one of ‘encourage customer to seek traditional medical care in addition to cannabis’ and/or ‘encourage customer to seek traditional medical care instead of cannabis.’ A 10-point increase in state medicalization score was associated with increased odds of respondents encouraging the customer to inform physician or other healthcare professional about cannabis use (adjusted odds ratio [aOR]=1.2, 95% confidence interval [CI] 1.0-1.4). This relationship was no longer significant when limited to non-clinicians (aOR=1.1, 95% CI 0.9-1.4). Working in a state with legalized adult-use cannabis was associated with increased odds of selecting ‘encourage customer to do additional research online’ when the sample was limited to non-clinicians (aOR=1.9, 95% CI 1.1-3.1, *p*= 0.02).

**Table 2:** Self-reported approach to customers with complex medical comorbidities

	All respondents (*n*=434)			Non-clinicians only (*n*=355)	
	Yes, n (%)	State medicalization score^a,b^ (aOR [95% CI])	Statewide adult use^a,b^ (aOR [95% CI])	State medicalization score^a,c^ (aOR [95% CI])	Statewide adult use^a,c^ (aOR [95% CI])
What do you do when you encounter a customer who is using cannabis to treat a medical condition (such as cancer, HIV/AIDS, or multiple sclerosis)?					
Encourage customer to inform physician or other healthcare professional about cannabis use	293 (67.5%)	**1.2 (1.0-1.4)**	1.2 (0.7-2.0)	1.1 (0.9-1.4)	1.3 (0.8-2.2)
Encourage customer to seek traditional medical care in addition to OR instead of cannabis	247 (56.9%)	1.0 (0.8-1.1)	1.3 (0.8-2.1)	1.0 (0.8-1.2)	1.3 (0.8-2.1)
Encourage customer to seek traditional medical care in addition to cannabis	216 (49.8%)	1.0 (0.9-1.1)	1.3 (0.8-2.0)	1.0 (0.8-1.1)	1.4 (0.8-2.3)
Encourage customer to do additional research online	212 (48.8%)	0.9 (0.8-1.0)	1.6 (1.0-2.6)	0.9 (0.8-1.1)	**1.9 (1.1-3.1)**
Encourage customer to seek traditional medical care instead of cannabis	47 (10.8%)	1.1 (0.8-1.4)	1.3 (0.5-3.5)	1.1 (0.8-1.6)	0.5 (0.2-1.9)
I don't do anything differently	42 (9.7%)	1.1 (0.8-1.3)	0.5 (0.2-1.1)	1.1 (0.8-1.3)	0.5 (0.2-1.2)
Encourage customer to continue only cannabis	40 (9.2%)	1.2 (0.9-1.6)	2.3 (0.9-6.0)	1.3 (1.0-1.8)	1.7 (0.6-5.1)
I have never encountered a customer who is using cannabis to treat a medical condition	10 (2.3%)	1.2 (0.7-1.9)	0.3 (0-2.2)	1.2 (0.7-1.9)	0.3 (0-2.2)

*Abbreviations*: *aOR* Adjusted Odds Ratio, *CI* confidence interval

^a^Regression full model includes: state medicalization score per 10-point increment; statewide adult use (yes/no [ref]); age per 10-point increment; role (budtender [ref], manager, physician/NP/PA, pharmacist, other); years working in cannabis industry (categorical- <6 months [ref], 6 months-1 year, 1-2 years, >2 years, no response); sales commission (yes/no); Education (categorical)

^b^ *n*=428, ^c^ *n*=351

^*^This table presents a series of mixed effects multivariable regression models in which each row represents the dependent variable with each column representing and independent variable in separate regression models. A random effect was included for state to account for clustering

Bolded values have *p*-value<0.05

‘Other, please describe’ is not shown

Participants’ self-reported approach to customers using cannabis to treat psychiatric co-morbidities such as depression, anxiety, or post-traumatic stress disorder are summarized in Supplementary Table 2a. Results were similar to findings for medical conditions. Most respondents indicated that they encourage customers to seek traditional medical care in addition to or instead of cannabis use (60.8%). Few reported that they have never encountered customers seeking cannabis for these conditions (0.5%). There was little evidence of any associations between state medicalization score or legalized adult use and responses to this question.

Participants’ self-reported approach to customers using cannabis for serious mental illnesses such as schizophrenia, bipolar disorder, and psychosis are summarized in Supplementary Table 3a. Results were similar to medical conditions and other psychiatric co-morbidities. Most respondents reported that they encourage customers to seek traditional medical care in addition to or instead of cannabis use (61.5%). Few reported that they have never encountered customers seeking cannabis for these conditions (6.9%). Working in a state with legalized adult-use cannabis was associated with increased odds of reporting ‘encourage customer to seek traditional medical/mental health care instead of cannabis’ (aOR=2.2, 95% CI 1.1-4.7, p=0.04). When limited to non-clinicians, legalized adult-use cannabis was associated with increased odds of reporting ‘I have never encountered a customer who is using cannabis to treat a serious mental illness’ (aOR=2.6, 95% CI 1.0-6.9, *p*=0.05).

Table 3 summarizes participants’ self-reported approach to customers who they suspect have CUD. Nearly half of respondents indicated that they have never encountered a customer who they suspected had CUD, and few indicated that they encourage those customers to see a health care professional. Higher state medicalization score was associated with decreased odds of reporting ‘discuss purchasing cannabis products that may help with the CUD (aOR=0.8, 95% CI 0.7-1.0, *p*=0.04); this association was no longer present when the sample was limited to non-clinicians.

**Table 3:** Self-reported approach to customers with suspected cannabis use disorder

	All respondents			Non-clinicians only	
	Yes, n(%) *n*=434	State medicalization score^a,b^ (aOR [95% CI])	Statewide adult use^a,b^ (aOR [95% CI])	State medicalization score^a,c^ (aOR [95% CI])	Statewide adult use^a,c^ (aOR [95% CI])
What do you do when you encounter a customer who you suspect has a cannabis use disorder?					
I have never encountered a customer who I suspected had cannabis use disorder	214 (49.3%)	1.1 (1.0-1.4)	1.1 (0.6-2.1)	1.1 (0.9-1.4)	1.0 (0.6-1.7)
Discuss purchasing cannabis products that may help with the cannabis use disorder	119 (27.4%)	**0.8 (0.7-1.0)**	0.7 (0.4-1.3)	0.9 (0.7-1.1)	0.7 (0.4-1.3)
I do not believe cannabis is addictive	114 (26.3%)	0.9 (0.8-1.1)	1.1 (0.6-1.9)	0.9 (0.7-1.1)	1.1 (0.6-2.0)
Refer to a physician or other healthcare professional	83 (19.1%)	0.9 (0.8-1.1)	0.6 (0.3-1.1)	0.9 (0.7-1.1)	0.5 (0.3-1.1)
I do not do anything differently for such customers	64 (14.7%)	0.9 (0.8-1.2)	2.0 (1.0-3.9)	1.0 (0.8-1.3)	2.0 (1.0-4.2)
I have never heard of cannabis use disorder	58 (13.4%)	1.0 (0.8-1.2)	1.5 (0.8-2.9)	1.0 (0.8-1.3)	1.8 (0.9-3.7)

*Abbreviations*: *aOR* Adjusted Odds Ratio, *CI* confidence interval

This table presents a series of mixed effects multivariable regression models in which each row represents the dependent variable with each column representing and independent variable in separate regression models. A random effect was included for state to account for clustering

^a^Regression full model includes: state medicalization score per 10-point increment; statewide adult use (yes/no [ref]); age per 10-point increment; role (budtender [ref], manager, physician/NP/PA, pharmacist, other); years working in cannabis industry (categorical- <6 months [ref], 6 months-1 year, 1-2 years, >2 years, no response); sales commission (yes/no); Education (categorical)

^b^ *n*=428, ^c^ *n*=351

Bolded values have *p*-value<0.05

‘Other, please describe’ is not shown

Table 4 summarizes responses to reasons why respondents have advised against cannabis purchase. Of the whole sample, 173 (39.9%) selected at least one of the reasons listed, excluding ‘other’. The most commonly selected reasons were pregnancy or nursing (19.4%), serious mental illness (17.7%), and appearing intoxicated (15.9%). The least commonly selected reasons were depression (2.3%), PTSD (2.3%), relationship problems (2.3%), withdrawal symptoms (2.8%), and difficulty keeping a job (4.6%). When limited to non-clinicians, higher state medicalization score was associated with decreased odds of advising against cannabis purchase for a customer having legal problems related to cannabis (aOR=0.7, 95% CI 0.5-1.0, *p*=0.04).

**Table 4:** Response to the prompt: Select all reasons why you have advised against cannabis purchase (*n*=434)

	All respondents	Non-clinicians only
Reason	n(%) (*n*=434)	n(%) (*n*=355)
Pregnancy or nursing	84 (19.4%)	62 (17.5%)
Serious mental illness (schizophrenia-bipolar disorder-psychosis)	77 (17.7%)	54 (15.2%)
Customer appeared intoxicated	69 (15.9%)	58 (16.3%)
Customer having legal problems related to cannabis	49 (11.3%)	39 (11%)
Customer having difficulty affording cannabis	39 (9%)	28 (7.9%)
Anxiety	27 (6.2%)	17 (4.8%)
Cognitive impairment (e.g.-dementia)	24 (5.5%)	19 (5.4%)
Customer needs more cannabis for the same effect	23 (5.3%)	20 (5.6%)
Customer was an older adult (age >65)	22 (5.1%)	12 (3.4%)
Customer having difficulty keeping a job	20 (4.6%)	18 (5.1%)
Customer has withdrawal symptoms	12 (2.8%)	9 (2.5%)
Depression	10 (2.3%)	6 (1.7%)
Post-traumatic stress disorder	10 (2.3%)	4 (1.1%)
Customer having relationship problem (e.g.-with partner or other close/family/friends)	10 (2.3%)	9 (2.5%)

‘Other, please describe’ is not shown

## Discussion

Our findings show that while state cannabis policies may regulate the quality of products sold in medical cannabis dispensaries, they were not associated with counseling provided by dispensary staff. State laws and regulations had minimal associations with the dispensing practices classified in this study, and when associations were found, they were inconsistent between conditions. Our findings highlight that cannabis dispensaries are not medical environments. When guidance on cannabis use is delegated to dispensary staff [9], health care professionals should not assume that their patients will be screened for contraindications to cannabis use, medication interactions with cannabinoids, or CUD. Thus, health care professionals should provide counseling on safe cannabis use, rather than leaving this task to dispensary staff. There is a growing list of existing resources health care professionals can use when providing such counseling [24–30].

Nearly half of participants reported that they had never encountered a customer with CUD, and more than a quarter reported not believing that cannabis is addictive. This finding was not associated with medicalization score or legalized adult-use cannabis, and this did not change when limited to non-clinicians. This is consistent with general public opinion that cannabis is less harmful than other substances including alcohol [31]. More than a quarter reported discussing the utility of cannabis products to treat CUD, which is not evidence-based [32]. It is notable that symptoms indicative of CUD, including tolerance, withdrawal, and relationship and work-related problems were seen as reasons to recommend against cannabis use in only 11% or fewer responses. While most dispensary staff reported they discuss involving healthcare professionals for medical and psychiatric comorbidities, far fewer referred individuals who had CUD to healthcare professionals.

CUD is associated with significant disability and impairment, and the number of individuals seeking out treatment for CUD have increased in recent years [33, 34]. Evidence-based treatments for CUD include cognitive behavioral therapy or contingency management [35]. Our findings highlight that customer-facing dispensary staff are not likely to identify CUD in individuals purchasing medical cannabis and that healthcare practitioners who know the patient are better fit to do so. It is important for customer-facing dispensary staff to not be the only voice giving recommendations to individuals purchasing cannabis.

Nearly 60% of dispensary staff reported they discuss involving healthcare professionals with their customers with medical and psychiatric comorbidities. This represents an opportunity for collaboration between dispensary staff and health care professionals. There is no standardization across states in the clinical and pharmacologic training required to provide recommendations as dispensary staff [36]. While dispensary staff may do their best to give appropriate recommendations to individuals, many have not been trained to do so while taking into consideration the complexities of medical and psychiatric comorbidities.

Findings regarding state medicalization score were inconsistent with our hypothesis that higher state medicalization score would be associated with recommending involving traditional health care professionals. Several factors could prevent medicalization scores from being associated with dispensary staff recommendations to consumers. Dispensary staff have no way of knowing whether individuals are already receiving advice on cannabis use from health care professionals [37], and may not know when health care professionals should be informed about cannabis use and when to recommend against cannabis use [36]. When health care professionals who recommend cannabis are not managing individual’s comorbidities, it may be difficult to advise the customer. It is not known how often these situations occur; however, analyses show that many individuals seeking medical and adult-use cannabis are doing so to manage clinical symptoms [4, 38, 39]. Alternative explanations for dispensary staff recommendations may also include regional or store-level variation not captured in this analysis.

Our study has limitations. Despite broad sampling, we recruited a modest sample size. It is unknown whether we recruited a representative proportion of clinically trained dispensary staff, and if having more clinically trained dispensary staff would have changed our findings. Further, multiple staff could respond from the same dispensary. Due to the complexity of tracking responses and anonymous nature of survey responses, we could not cluster by those who were from the same dispensary. Additionally, dispensary lists may be obsolete due to dispensaries opening and closing, such that a denominator for response rates cannot be determined. Our study was administered by an identifiable academic center, making findings vulnerable to social desirability bias; respondents may have documented the ‘right’ answer rather than what they do in practice. Future qualitative studies of dispensary staff would improve our understanding of the barriers and facilitators to integrating traditional health care professionals with dispensary recommendations and the motivations for dispensary staff’s recommendations.

## Supplementary Information

**Additional file 1.** 

## Data Availability

The datasets used and/or analyzed during the current study are not publicly available due to concerns about protecting participants’ personal information, but de-identified data are available from the corresponding author on reasonable request.

## Abbreviations

CUD: Cannabis use disorder
SD: Standard deviation
OR: Odds ratio
THC: delta-9-tetrahydrocannabinol
