# Supplementary material for: Cannabis dispensary staff approaches to counseling on potential contraindications to cannabis use: insights from a national self-report survey
Source: BMC Prim Care. 2023 Jul 14;24:145. doi: 10.1186/s12875-023-02095-5 (PMC10347704; doi:10.1186/s12875-023-02095-5)
Supplement: Supplementary file 1 — Additional file 1. [file 12875_2023_2095_MOESM1_ESM.docx]

Supplementary Materials

| **Table 1A. Survey questions and potential responses** | |
| --- | --- |
| *What do you do when you encounter a customer who is using cannabis to treat a medical condition (such as cancer, HIV/AIDS, or multiple sclerosis)? (select all that apply)* | |
|  | Encourage customer to seek traditional medical care in addition to cannabis |
|  | Encourage customer to seek traditional medical care instead of cannabis |
|  | Encourage customer to inform physician or other healthcare professional about cannabis use |
|  | Encourage customer to continue only cannabis |
|  | Encourage customer to do additional research online |
|  | I don't do anything differently |
|  | I have never encountered a customer who is using cannabis to treat a medical condition |
|  | Other, please describe |
|  | Encourage customer to seek traditional medical care in addition to OR instead of cannabis |
| *What do you do when you encounter a customer who is using cannabis to treat depression, anxiety, or post-traumatic stress disorder? (select all that apply)* | |
|  | Encourage customer to seek traditional medical/mental health care in addition to cannabis |
|  | Encourage customer to seek traditional medical/mental health care instead of cannabis |
|  | Encourage customer to inform physician or other healthcare professional about cannabis use |
|  | Encourage customer to do additional research online |
|  | I don't do anything differently |
|  | Other, please describe |
|  | Encourage customer to seek traditional medical/mental health care in addition to OR instead of cannabis |
| *What do you do when you encounter a customer who is using cannabis to treat a serious mental illness (such as schizophrenia, bipolar disorder, or psychosis)? (select all that apply)* | |
|  | Encourage customer to seek traditional medical/mental health care in addition to cannabis |
|  | Encourage customer to seek traditional medical/mental health care instead of cannabis |
|  | Encourage customer to inform physician or other healthcare professional about cannabis use |
|  | Encourage customer to do additional research online |
|  | I don't do anything differently |
|  | I have never encountered a customer who is using cannabis to treat serious mental illness |
|  | Other, please describe |
|  | Encourage customer to seek traditional medical/mental health care in addition to OR instead of cannabis |
| *What do you do when you encounter a customer who you suspect has a cannabis use disorder? (select all that apply)* | |
|  | Refer to a physician or other health professional |
|  | Discuss purchasing cannabis products that may help with the cannabis use disorder |
|  | I do not do anything differently for such customers |
|  | I have never encountered a customer who I suspected had cannabis use disorder |
|  | I have never heard of cannabis use disorder |
|  | I do not believe cannabis is addictive |
|  | Other, please describe |
| *Select all reasons why you have advised against cannabis purchase* | |
|  | Pregnancy or nursing |
|  | Serious mental illness (schizophrenia-bipolar disorder-psychosis) |
|  | Customer appeared intoxicated |
|  | Customer having legal problems related to cannabis |
|  | Customer having difficulty affording cannabis |
|  | Anxiety |
|  | Cognitive impairment (e.g.-dementia) |
|  | Customer needs more cannabis for the same effect |
|  | Customer was an older adult (age >65) |
|  | Customer having difficulty keeping a job |
|  | Customer has withdrawal symptoms |
|  | Depression |
|  | Post-traumatic stress disorder |
|  | Customer having relationship problem (e.g.-with partner or other close/family/friends) |

| **Table 2a**. Self-reported approach to customers with psychiatric comorbidities | | | | | | |
| --- | --- | --- | --- | --- | --- | --- |
|  |  | All respondents | | | Non-clinicians only | |
|  |  | Yes, n(%)  n=434 | State medicalization score^a,b^  (aOR [95% CI]) | Statewide adult use^a,b^  (aOR [95% CI]) | State medicalization score^a,c^  (aOR [95% CI]) | Statewide adult use^a,c^  (aOR [95% CI]) |
| What do you do when you encounter a customer who is using cannabis to treat depression, anxiety, or post-traumatic stress disorder? | | | | | | |
|  | Encourage customer to seek traditional medical/mental health care in addition to OR instead of cannabis | 264 (60.8%) |  |  |  |  |
|  | Encourage customer to inform physician or other healthcare professional about cannabis use | 259 (59.7%) | 1.0 (0.9-1.2) | 1.3 (0.8-2.1) | 1.0 (0.9-1.2) | 1.3 (0.8-2.2) |
|  | Encourage customer to seek traditional medical/mental health care in addition to cannabis | 226 (52.1%) | 1.0 (0.9-1.2) | 1.3 (0.8-2.2) | 1.0 (0.9-1.2) | 1.4 (0.8-2.3) |
|  | Encourage customer to do additional research online | 220 (50.7%) | 0.9 (0.8-1.0) | 1.4 (0.9-2.3) | 0.9 (0.7-1.1) | 1.7 (1.0-2.9) |
|  | Encourage customer to seek traditional medical/mental health care instead of cannabis | 60 (13.8%) | 1.1 (0.8-1.4) | 1.6 (0.6-4.0) | 1.1 (0.7-1.6) | 1.3 (0.4-4.2) |
|  | I don't do anything differently | 56 (12.9%) | 1.2 (0.9-1.4) | 0.7 (0.3-1.5) | 1.2 (1.0-1.5) | 0.6 (0.3-1.4) |
|  | I have never encountered a customer who is using cannabis to treat one of these disorders | 2 (0.5%) |  |  |  |  |
| Abbreviations: aOR- Adjusted Odds Ratio; CI- confidence interval;  ^a^Regression full model includes: state medicalization score per 10-point increment; statewide adult use (yes/no [ref]); age per 10-point increment; role (budtender [ref], manager, physician/NP/PA, pharmacist, other); years working in cannabis industry (categorical- <6 months [ref], 6 months-1 year, 1-2 years, >2 years, no response); sales commission (yes/no); Education (categorical)  ^b^n=428, ^c^n=351  * This table presents a series of mixed effects multivariable regression models in which each row represents the dependent variable with each column representing and independent variable in separate regression models. A random effect was included for state to account for clustering.  Bolded values have p-value<0.05  ‘Other, please describe’ is not shown | | | | | | |

| **Table 3a**. Self-reported approach to customers with serious mental illness | | | | | | |
| --- | --- | --- | --- | --- | --- | --- |
|  |  | All respondents | | | Non-clinicians only | |
|  |  | Yes, n(%)  n=434 | State medicalization score^a,b^  (aOR [95% CI]) | Statewide adult use^a,b^  (aOR [95% CI]) | State medicalization score^a,c^  (aOR [95% CI]) | Statewide adult use^a,c^  (aOR [95% CI]) |
| What do you do when you encounter a customer who is using cannabis to treat a serious mental illness (such as schizophrenia, bipolar disorder, or psychosis)? | | | | | | |
|  | Encourage customer to seek traditional medical/mental health care in addition to OR instead of cannabis | 267 (61.5%) | 1.1 (0.9-1.2) | 1.6 (1.0-2.6) | 1.0 (0.8-1.2) | 1.3 (0.8-2.2) |
|  | Encourage customer to inform physician or other healthcare professional about cannabis use | 259 (59.7%) | 1.0 (0.8-1.1) | 0.7 (0.5-1.2) | 1.0 (0.8-1.1) | 0.8 (0.5-1.3) |
|  | Encourage customer to seek traditional medical/mental health care in addition to cannabis | 211 (48.6%) | 1.0 (0.9-1.2) | 1.2 (0.7-1.8) | 0.9 (0.8-1.1) | 1.2 (0.7-2.0) |
|  | Encourage customer to do additional research online | 179 (41.2%) | 0.9 (0.7-1.0) | 1.1 (0.7-1.8) | 0.9 (0.7-1.0) | 1.4 (0.8-2.3) |
|  | Encourage customer to seek traditional medical/mental health care instead of cannabis | 77 (17.7%) | 1.2 (0.9-1.5) | **2.2 (1.1-4.7)** | 1.2 (0.9-1.6) | 1.3 (0.6-3.1) |
|  | I don't do anything differently | 38 (8.8%) | 1.0 (0.7-1.3) | 0.7 (0.3-1.7) | 1.0 (0.8-1.3) | 0.8 (0.3-1.9) |
|  | I have never encountered a customer who is using cannabis to treat serious mental illness | 30 (6.9%) | 1.0 (0.8-1.4) | 2.3 (0.9-5.9) | 1.1 (0.8-1.5) | **2.6 (1.0-6.9)** |
| Abbreviations: aOR- Adjusted Odds Ratio; CI- confidence interval;  ^a^Regression full model includes: state medicalization score per 10-point increment; statewide adult use (yes/no [ref]); age per 10-point increment; role (budtender [ref], manager, physician/NP/PA, pharmacist, other); years working in cannabis industry (categorical- <6 months [ref], 6 months-1 year, 1-2 years, >2 years, no response); sales commission (yes/no); Education (categorical)  ^b^n=428, ^c^n=351  *This table presents a series of mixed effects multivariable regression models in which each row represents the dependent variable with each column representing and independent variable in separate regression models. A random effect was included for state to account for clustering.  Bolded values have p-value<0.05  ‘Other, please describe’ is not shown | | | | | | |

| **Table 4a**. Response to the prompt: Select all reasons why you have advised against cannabis purchase and associations with state medicalization score and legalized adult-use cannabis (n=434) | | | | | | |
| --- | --- | --- | --- | --- | --- | --- |
|  | All respondents | | | Non-clinicians only | | |
| Reason | n(%)  (n=434) | State medicalization score^a,b^  (aOR [95% CI]) | Statewide adult use^a,b^  (aOR [95% CI]) | n(%)  n=355 | State medicalization score^a,c^  (aOR [95% CI]) | Statewide adult use^a,c^  (aOR [95% CI]) |
| Pregnancy or nursing | 84 (19.4%) | 1.0  (0.7-1.4) | 1.0  (0.4-2.7) | 62 (17.5%) | 0.9  (0.7-1.3) | 1.1  (0.4-2.7) |
| Serious mental illness (schizophrenia-bipolar disorder-psychosis) | 77 (17.7%) | 1.2  (0.9-1.5) | 1.2  (0.5-2.9) | 54 (15.2%) | 1.2  (0.9-1.6) | 1.3  (0.5-3.1) |
| Customer appeared intoxicated | 69 (15.9%) | 1.2  (0.8-1.9) | 1.5  (0.5-5.4) | 58 (16.3%) | 1.0  (0.7-1.6) | 1.6  (0.5-5.4) |
| Customer having legal problems related to cannabis | 49 (11.3%) | 0.8  (0.7-1.1) | 1.0  (0.5-2.0) | 39 (11%) | 0.7  (0.5-1.0) | 1.0  (0.4-2.2) |
| Customer having difficulty affording cannabis | 39 (9%) | 0.8  (0.6-1.1) | 1.6  (0.7-3.7) | 28 (7.9%) | 0.6  (0.3-1.2) | 2.2  (0.5-9.3) |
| Anxiety | 27 (6.2%) | 0.8  (0.6-1.2) | 0.4  (0.1-1.5) | 17 (4.8%) | 0.9  (0.6-1.3) | 0.4  (0.1-1.4) |
| Cognitive impairment (e.g.-dementia) | 24 (5.5%) | 1.0  (0.7-1.3) | 1.1  (0.4-2.9) | 19 (5.4%) | 0.7  (0.5-1.1) | 1.1  (0.4-3.2) |
| Customer needs more cannabis for the same effect | 23 (5.3%) | 0.8  (0.5-1.2) | 1.3  (0.4-4.6) | 20 (5.6%) | 0.8  (0.5-1.3) | 1.7  (0.5-5.3) |
| Customer was an older adult (age >65) | 22 (5.1%) | 0.8  (0.6-1.3) | 1.4  (0.4-4.4) | 12 (3.4%) | 0.5  (0.2-1.0) | 1.6  (0.4-6.0) |
| Customer having difficulty keeping a job | 20 (4.6%) | 0.7  (0.5-1.1) | 0.8  (0.3-2.4) | 18 (5.1%) | 0.6  (0.4-1.0) | 0.8  (0.3-2.4) |
| Customer has withdrawal symptoms | 12 (2.8%) | 0.7  (0.4-1.1) | 1.1  (0.3-4.5) | 9 (2.5%) | 0.6  (0.3-1.2) | 2.4  (0.5-11.5) |
| Depression | 10 (2.3%) | 0.6  (0.3-1.2) | 1.1  (0.2-7.0) | 6 (1.7%) | 0.6  (0.3-1.3) | 0.9  (0.1-6.6) |
| Post-traumatic stress disorder | 10 (2.3%) | 0.8  (0.5-1.4) | 0.3  (0-2.4) | 4 (1.1%) |  |  |
| Customer having relationship problem (e.g.-with partner or other close/family/friends) | 10 (2.3%) | 0.5  (0-68.5) |  | 9 (2.5%) | 0.7  (0.1-4.2) | 0.1  (0-70.7) |
| Abbreviations: aOR- Adjusted Odds Ratio; CI- confidence interval;  ^a^Regression full model includes: state medicalization score per 10-point increment; statewide adult use (yes/no [ref]); age per 10-point increment; role (budtender [ref], manager, physician/NP/PA, pharmacist, other); years working in cannabis industry (categorical- <6 months [ref], 6 months-1 year, 1-2 years, >2 years, no response); sales commission (yes/no); Education (categorical)  ^b^n=428, ^c^n=351  * This table presents a series of mixed effects multivariable regression models in which each row represents the dependent variable with each column representing and independent variable in separate regression models. A random effect was included for state to account for clustering.  Bolded values have p-value<0.05  ‘Other, please describe’ is not shown | | | | | | |
